# Multitrophic Effects of Belowground Parasitoid Learning

**DOI:** 10.1038/s41598-017-02193-2

**Published:** 2017-05-18

**Authors:** Denis S. Willett, Hans T. Alborn, Lukasz L. Stelinski

**Affiliations:** 1 Agricultural Research Service, United States Department of Agriculture, Center for Medical, Agricultural and Veterinary Entomology, Gainesville, FL 32608 USA; 2 University of Florida, Entomology and Nematology Department, Citrus Research and Education Center, Lake Alfred, FL 33950 USA

## Abstract

The ability to learn allows organisms to take advantage of dynamic and ephemeral opportunities in their environment. Here we show that learning in belowground entomopathogenic nematodes has cascading multitrophic effects on their hosts, other nematodes, and nematophagous fungal predators. In addition to quantifying these effects, we show that social behavioral plasticity in these belowground parasitoids can amplify signaling by plant defense pathways and results in an almost doubling of insect herbivore infection by entomopathogenic nematodes. Cumulatively, these effects point to the critical role of plant signaling in regulating community structure while suggesting an equally important role for behavioral plasticity in shaping community dynamics.

## Introduction

Social learning in higher organisms is thought to hold adaptive significance^1^. Organisms with high behavioral plasticity, the ability to modify behavior based on past experiences, can avail themselves of dynamic ephemeral opportunities for enhancing fitness that would not exist if genetic inheritance were solely responsible for adaption^1^. Primates, for example, benefit by taking advantage of resources made available from socially learned tool use^2, 3^. Similarly, New Caledonian crows benefit from social transmission of tool designs that facilitate access to otherwise unavailable food resources^4^. While behavioral plasticity, especially social behavioral plasticity, has direct fitness benefits for the organism responding to ephemeral environmental cues, the ability of one individual or species to modify behavior based on past experiences may have cascading impacts on other trophic levels in a community.

Evidence from parasitoids can be used to begin to elucidate the cascading trophic effects of social learning^5^. In contrast to a general predator-prey relationship where many predators affect multiple prey organisms, host-parasitoid relationships tend to be highly specific^5, 6^. In a given system, the primary goal of a parasitoid is to find (often) a single specific host^5, 6^. Likewise, hosts must (often) avoid a single specific parasitoid^5, 6^. This unique dynamic sets the stage for an arms race where any ability to take advantage of ephemeral opportunities in a complex environment has immediate consequences for a tightly linked system.

For parasitoids, this advantage is often behavioral plasticity. Parasitoids rely on learned search images to better identify and locate their hosts^5, 6^. These images need not be merely visual; search images can also be auditory^7^ or olfactory. For example, olfactory cues are frequently used by insect and entomopathogenic nematode parasitoids to find their host^6, 8, 9^. While entomopathogenic nematodes, which infect and kill insect larvae belowground with the help of endosymbiotic bacteria, have differences with aboveground insect parasitoids^10, 11^, their patterns of host infection and associated host mortality fit the functional definition of a parasitoid^12^. Indeed, there are similarities between the two. Aboveground, specific blends of herbivore induced plant volatiles recruit parasitic wasps^13^. Belowground, volatile compounds released by plants after induction of defense pathways recruit entomopathogenic nematodes. In maize, feeding belowground by larvae of the beetle *Diabrotica virgifera virgifera* induces release of (*E*)-*β* caryophyllene^14^ which recruits entomopathogenic nematodes. In certain citrus cultivars, induction of plant defense pathways through belowground herbivory by *Diaprepes abbreviatus* weevil larvae or foliar application of methyl salicylate induces belowground release of pregeijerene and d-limonene respectively^15–18^.

**Figure 1:**
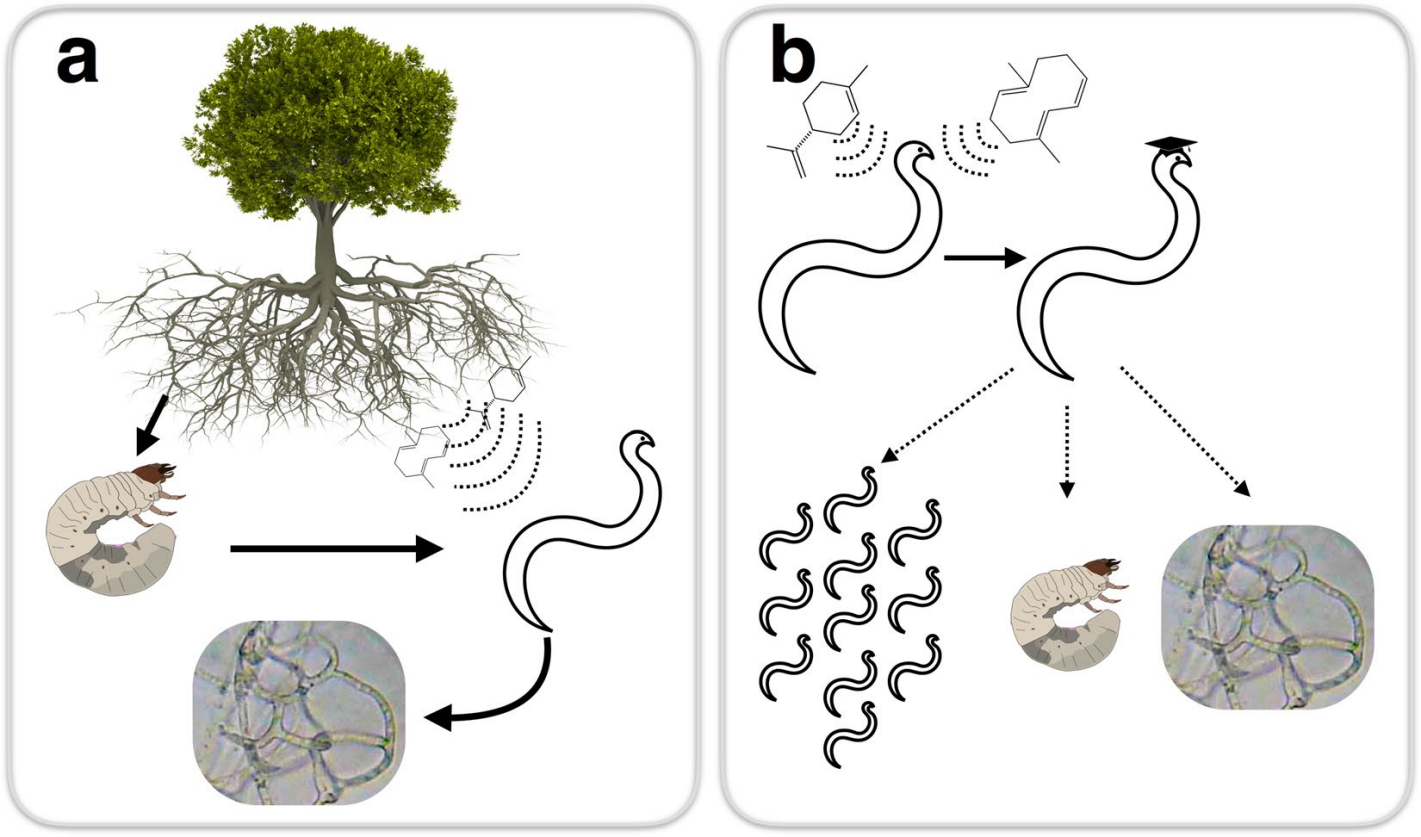
Belowground Multitrophic Interactions. (**a**) In the field, citrus roots are fed upon by larvae of the weevil *D*. *abbreviatus*. Entomopathogenic nematodes, parasitoids of insect larvae, respond to volatile terpene compounds released by roots following stimulation of plant defenses. Nematophagous fungi capture and consume entomopathogenic nematodes. (**b**) To determine the multitrophic effects of belowground parasitoid learning, entomopathogenic nematodes were exposed to volatiles they encounter in a belowground environment. These exposed, ‘educated’, entomopathogenic nematodes were then assayed to determine effects on other entomopathogenic nematodes, host insects, and nematophagous fungi. Citrus tree ©Can Stock Photo Inc. Insect larva courtesy BugBoy 52.40. Fungal hyphae courtesy Bob Blaylock.

Because many parasitoids rely on responses to plant volatiles to find their hosts above and belowground, the resulting parasitoid-host interactions are often inherently multitrophic. While behavioral plasticity in parasitoids has been shown to affect population dynamics of host species^19^, extended multitrophic consequences of parasitoid learning have not been quantified. Here, we use a belowground model system involving plant volatiles, entomopathogenic nematodes (parasitoids of insect larvae), their hosts, and nematophagous fungi (predators of the nematode parasitoids) to understand the cascading consequences of parasitoid social behavioral plasticity throughout the system (Fig. 1).

To quantify these consequences at multiple trophic levels, we first quantify the effect of behavioral plasticity in entomopathogenic nematodes on their natural host, larvae of the weevil *Diaprepes abbreviatus*. Second, we assess how behavioral plasticity in entomopathogenic nematodes can affect infection of other potential hosts. Third, we examine effects of entomopathogenic nematode behavioral plasticity on predation by nematophagous fungi. Fourth, we highlight how social behavioral plasticity can result in cross-species transfer of experience and affect host infection. Finally, we combine individual effects into a multitrophic assessment of cascading impacts of belowground parasitoid learning.

## Results

### Host Effects

To quantify host effects of parasitoid behavioral plasticity, infection of *D*. *abbreviatus* larvae by four species of entomopathogenic nematodes across two genera (*Steinernema diaprepesi*, *S*. *riobrave*, *S*. *carpocapsae*, and *Heterorhabditis indica*) was monitored in sand-filled bioassays. Entomopathogenic nematodes infect and kill insect larvae with the help of endosymbiotic bacteria and can be effective and efficient biological control agents^20–22^. Two of the entomopathogenic nematode species used (*S*. *diaprepesi* and *H*. *indica*) are commonly found infecting *D*. *abbreviatus* larvae in Florida citrus orchards^23^ where they inhabit a complex and dynamic belowground environment punctuated by the presence of transient plant volatile signals such as d-limonene, (*E*)-*β* caryophyllene, and pregeijerene^15–18^. Cohorts of these entomopathogenic nematode infective juveniles were exposed for 72 hours to either blank controls or one of either d-limonene, (*E*)-*β* caryophyllene, or pregeijerene. After exposure to the plant volatiles, nematode cohorts were then introduced to four-choice, sand-filled olfactometers and given the opportunity to infect *D*. *abbreviatus* larval hosts paired with one of the volatiles to which the cohorts had been exposed: either a blank control, d-limonene, (*E*)-*β* caryophyllene or pregeijerene. Host infection was then modeled using logistic regression to determine infection probabilities under different learning regimes.

**Figure 2:**
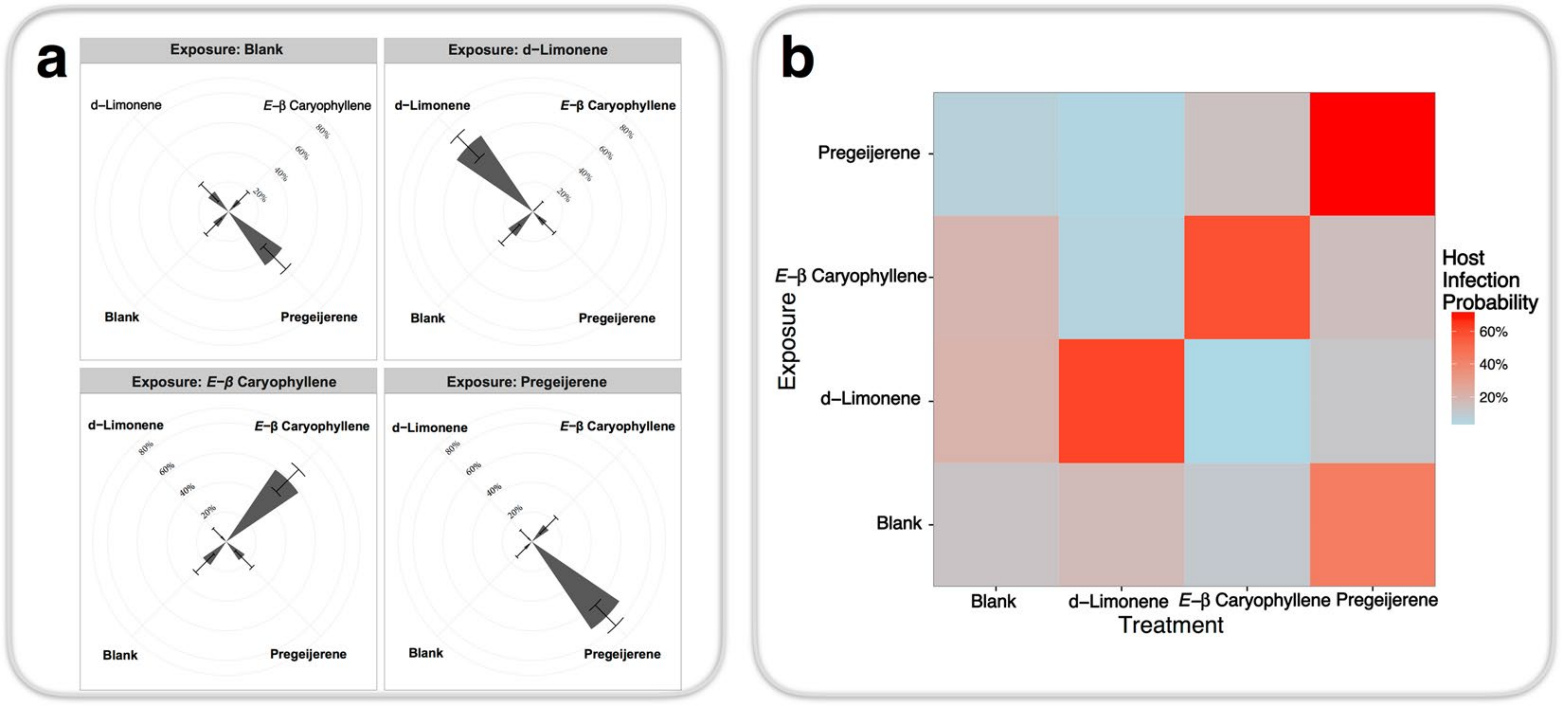
Parasitoid Learning Affects Host Infection. Prior exposure to belowground volatiles affects entomopathogenic nematode infection of larvae of the weevil *D*. *abbreviatus*, a natural host. (**a**) Cohorts of entomopathogenic nematode infective juveniles were exposed to blank controls and three common belowground plant volatiles then assayed in four-arm, sand-filled olfactometers where they responded to *D*. *abbreviatus* larvae paired with those volatiles. Concentric circles indicate host infection probability. Wedges and error bars denote host infection probability and ninety-five percent confidence intervals respectively. (**b**) Prior exposure to belowground volatiles increases host infection probability when host is paired with exposed volatile.

Behavioral plasticity in response to exposure to plant volatiles affected infection of *D*. *abbreviatus* larvae (Fig. 2). Both treatment and the interaction between treatment and exposure had a significant effect (Treatment: *df* = 3, *χ* ^2^ = 90.0, *P* < 0.001; Interaction: *df* = 12, *χ* ^2^ = 281.3, *P* < 0.001, analysis of deviance) on probability of host infection. With no exposure (i.e. exposure to blank control not containing plant volatiles), host infection probabilities were significantly greater (*P* < 0.001, Dunnett’s test) for larvae paired with the highly attractive herbivore induced plant volatile pregeijerene. Infection probabilities for hosts paired with pregeijerene were 15.5% [95% CI: 6.6%, 32.4%] greater than blank controls, 20.2% [9.4%, 38.2%] greater than d-limonene, and 12.5% [4.9%, 28.5%] greater than (*E*)-*β* caryophyllene. Exposure to plant volatiles resulted in significant increases (d-Limonene: 91.1% [83.8%, 95.3%], *P* < 0.001; (*E*)-*β* caryophyllene: 90.0% [81.7%, 94.7%], *P* < 0.001; pregeijerene: 84.4% [75.5%, 90.5%], *P* < 0.001) in host infection probability when the host was paired with that volatile. All nematode species behaved similarly; there was no significant effect of nematode species (*df* = 3, *χ* ^2^ = 1.1, *P* = 0.78).

### Additional Host Effects

Entomopathogenic nematodes may be somewhat unique in that they have the potential to infect a wide range of insect larvae that may not be considered traditional hosts. To investigate additional host effects of parasitoid behavioral plasticity, infection of Caribbean fruit fly *Anastrepha suspensa*, mealworm *Tenebrio molitor*, and waxworm *Galleria mellonella* larvae was evaluated alongside infection of *D*. *abbreviatus* to cohorts of *H*. *indica* infective juveniles exposed to d-limonene or blank controls in four-arm, sand-filled olfactometers as above.

**Figure 3:**
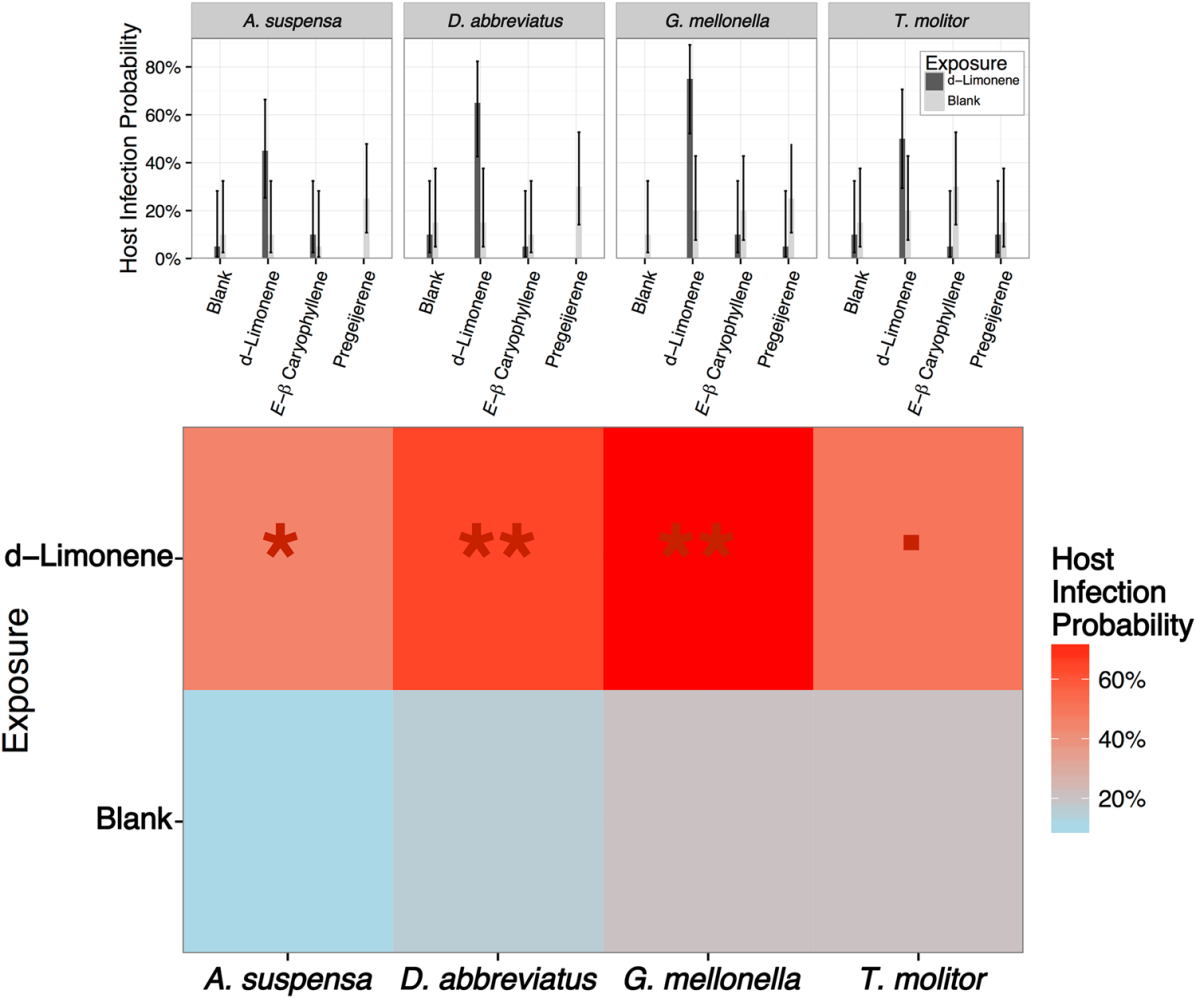
Parasitoid Learning Affects Alternate Host Infection. Prior exposure to d-limonene increases host infection probability for the natural host, *D*. *abbreviatus*, the host in which it is reared, *G*. *mellonella*, and the caribbean fruit fly *A*. *suspensa*. Cohorts of entomopathogenic nematode *H*. *indica* infective juveniles were exposed to blank controls or d-limonene then assayed in four-arm, sand-filled olfactometers where they responded to larvae paired with three common plant volatiles. Bars and error bars denote host infection probability and ninety-five percent confidence intervals respectively. Double asterisks denotes significant enhancement of infection probability at *P* < 0.01. A single asterisk denotes significant enhancement of infection probability at *P* < 0.05. A period denotes marginally significant enhancement of infection probability at *P* < 0.1.

Behavioral plasticity in response to exposure to d-limonene resulted in increased infections of all hosts (Fig. 3). While prior exposure to d-limonene resulted in significantly increased infection probability of *D*. *abbreviatus* (91.3% [69.4%, 98.0%], *P* = 0.003, Tukey’s test) as in the host effects trials above, prior exposure to d-limonene also significantly increased infection probability of Caribbean fruit fly larvae (by 88.0% [57.2%, 97.6%], *P* = 0.02, Tukey’s test) and waxworm larvae (by 92.3% [73.0%, 98.2%], *P* = 0.001, Tukey’s test) over exposure to blank controls. Prior exposure to d-limonene marginally increased infection probability of mealworm larvae (by 80.0% [49.6%, 94.2%], *P* = 0.05, Tukey’s test).

### Predator Effects

In addition to procuring host insects, entomopathogenic nematode parasitoids must contend with a variety of predators eager for a quick meal. To investigate effects of behavioral plasticity in entomopathogenic nematodes on their nematophagous fungal predators, cohorts of entomopathogenic nematode *H*. *indica* infective juveniles were exposed either to d-limonene or blank controls then introduced to four-choice bioassays which were either inoculated with the nematode-trapping fungus *Arthrobotrys dactyloides* ^24^ or with blank controls. After passing through the nematophagous fungal (or a control) guantlet, infective juveniles had the opportunity to infect insect larvae paired with either d-limonene, (*E*)-*β* caryophyllene, pregeijerene, or a blank control.

**Figure 4:**
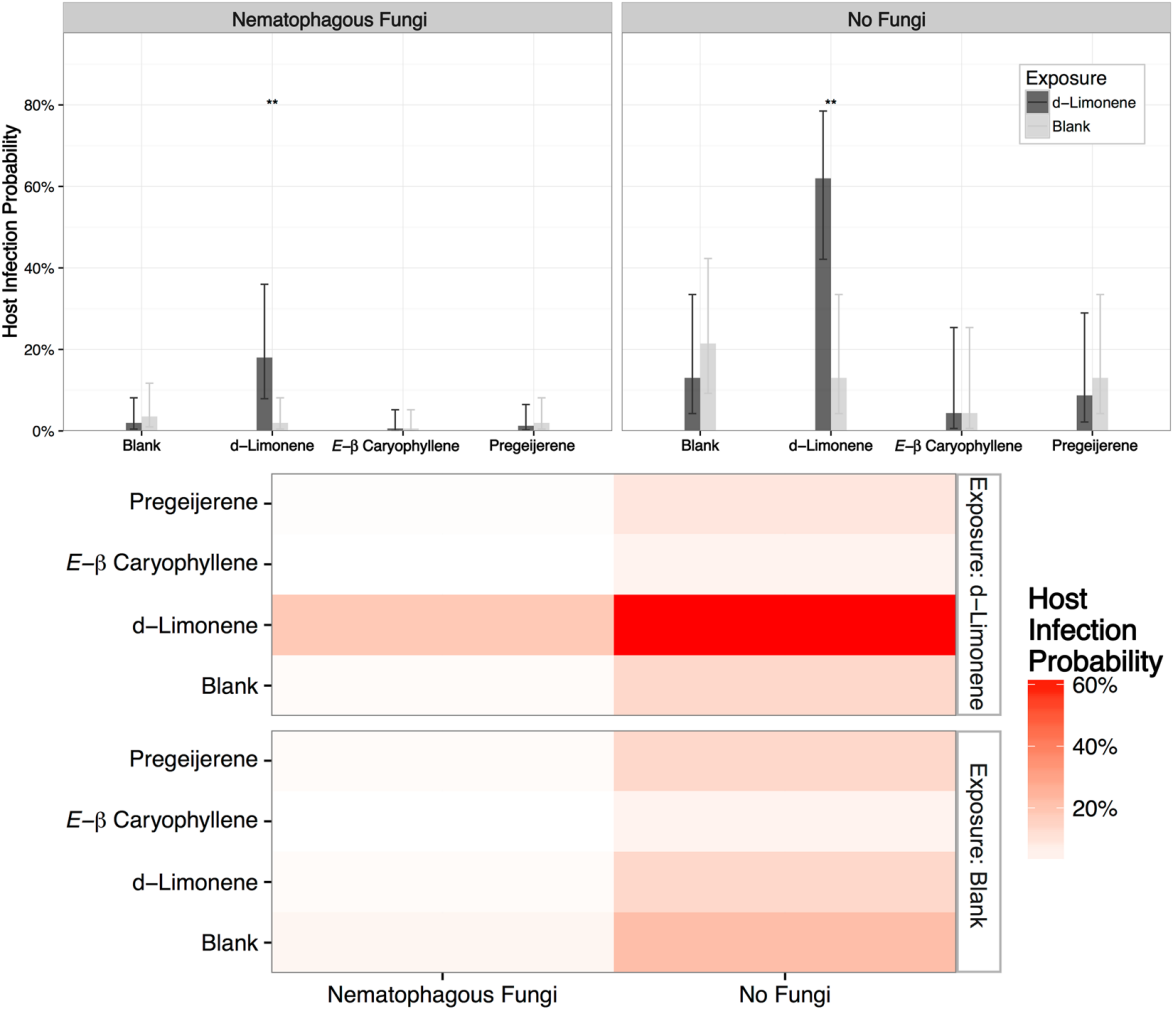
Parasitoid Learning Affects Predation. Prior exposure to d-limonene increases host infection probability despite predation by nematophagous fungi. Cohorts of entomopathogenic nematode *H*. *indica* infective juveniles were exposed to blank controls or d-limonene then assayed in the presence and absence of the nematophagous fungal predator *A*. *dactyloides*. Bars and error bars denote host infection probability and ninety-five percent confidence intervals respectively. Double asterisks denotes significant enhancement of infection probability at *P* < 0.01.

The presence of nematophagous fungi significantly (*P* = 0.0001) altered host infection probabilities reducing nonexposed nematode host infection probabilities by 88.1% [73.1%, 95.3%] to essentially zero (lower 95% confidence level of 0.07%) (Fig. 4). Exposure to d-limonene reversed this trend significantly (*P* = 0.001, Tukey’s test) increasing host infection probabilities by 91.6% [72.5%, 97.8%] to 18.1% [8.0%, 36.0%] despite the nematophagous fungal gauntlet.

### Host Effects of Social Parasitoid Learning

Entomopathogenic nematodes spend much of their life in close proximity to other entomopathogenic nematodes and rely on group attack to overcome host immune systems^20, 25^. Additionally, entomopathogenic nematodes can demonstrate social behavioral plasticity^26^. Cohorts of experienced entomopathogenic nematodes can influence the behavior of non-experienced cohorts through what is thought to be a follow the leader dynamic^26, 27^. To investigate host effects of social behavioral plasticity in entomopathogenic nematodes, cohorts of 50 *H*. *indica* infective juveniles were exposed to either d-limonene or blank controls. Following exposure, those cohorts of 50 nematodes (the ‘leaders’) were then combined with cohorts of 2450 nonexposed infective juveniles (the ‘followers’) of either *H*. *indica*, *S*. *diaprepesi*, or *S*. *riobrave*. These combined groups were then introduced to four-arm, sand-filled olfactometers where they had the opportunity to recruit to *D*. *abbreviatus* larvae paired with either a blank control, d-limonene, (*E*)-*β* caryophyllene or pregeijerene. Fifty entomopathogenic nematodes (assuming they all attempted host infection) are often not enough to overcome host immune defenses^25^. Indeed, host infection probability from cohorts of 50 entomopathogenic nematodes is just 1.0% [0.1%, 6.9%].

**Figure 5:**
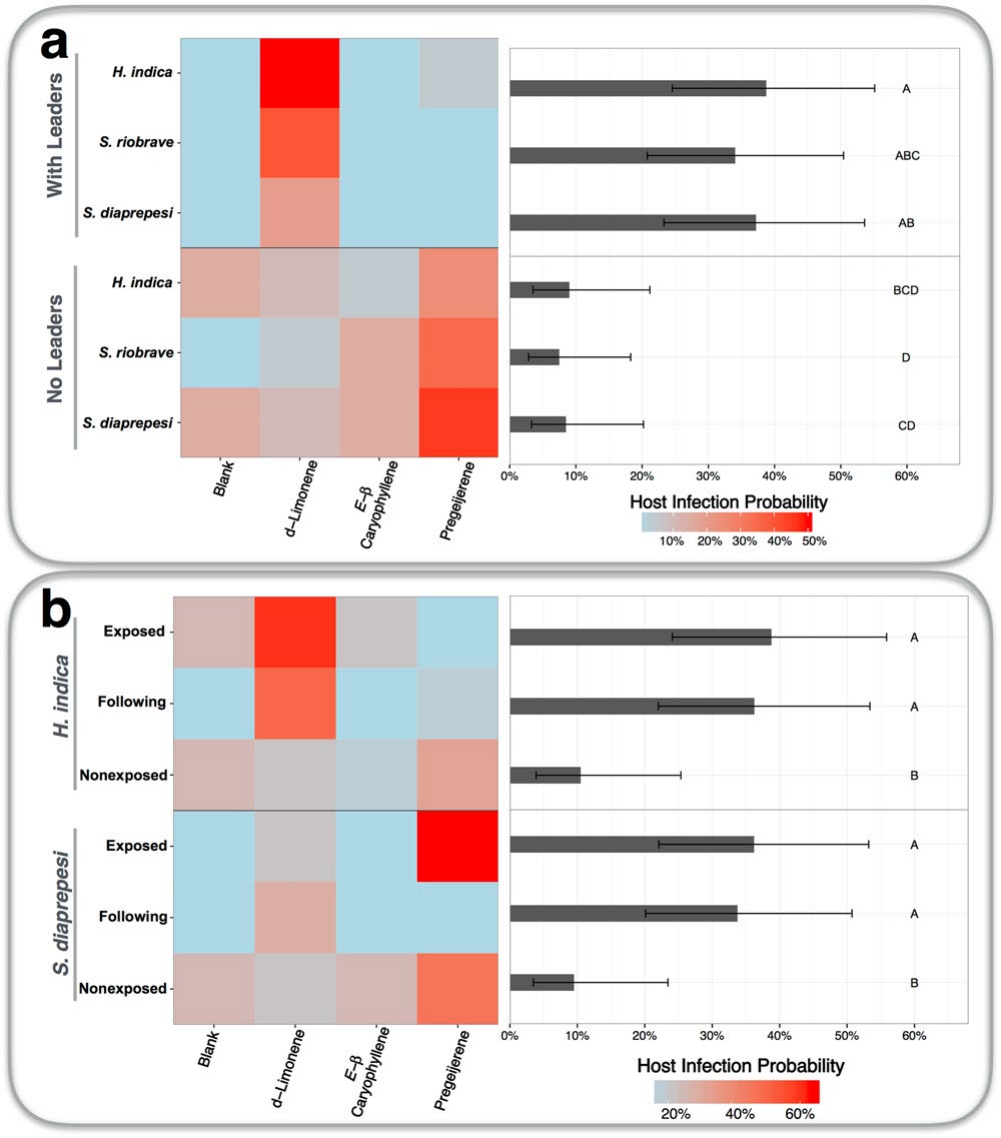
Social Behavioral Plasticity in Parasitoids affects Host Infection. (**a**) Cohorts of entomopathogenic infective juveniles exposed to d-limonene can influence infective juveniles of other species resulting in increased host infection probability. For trials with leaders, cohorts of 50 *H*. *indica* infective juveniles exposed to d-limonene (the ‘leaders’) were paired with 2450 nonexposed infective juveniles of either *H*. *indica*, *S*. *riobrave*, or *S*. *diaprepesi*. For trials without leaders, cohorts consisted of 2500 nonexposed infective juveniles. (**b**) Mixed leader-follower cohorts mimic performance of exposed only cohorts. Exposed cohorts consisted of 2500 infective juveniles exposed to d-limonene. Following cohorts consisted of mixed groups of 50 *H*. *indica* infective juveniles paired with 2450 nonexposed infective juveniles. Nonexposed cohorts consisted of 2500 infective juveniles exposed to blank controls not consisting of plant volatiles. Bars and error bars denote mean infection probability of *D*. *abbreviatus* larvae paired with d-limonene and 95% confidence intervals respectively. Groups not sharing letters are significantly different (*P* < 0.05, Tukey’s test).

Cohorts of 50 educated nematodes are able, however, to influence other entomopathogenic nematodes. When numbers of responding infective juveniles were increased to 2500, infection probabilities of *D*. *abbreviatus* larvae paired with d-limonene significantly (*P* = 0.0007, Tukey’s test) increased by 97.2% [81.7%, 99.6%]. Joining cohorts of 50 *H*. *indica* infective juveniles that were exposed to d-limonene with 2450 nonexposed infective juveniles of either *H*. *indica*, *S*. *riobrave*, or *S*. *diaprepesi* further increased infection probability of *D*. *abbreviatus* paired with d-limonene by 13.5% [5.2%, 31.1%] (Fig. 5a). In fact, differences between infection probabilities by 2500 infective juveniles, all of whom were exposed to d-limonene and infections probabilities by combined 50 leader and 2450 follower groups were not significantly different (*P* > 0.05, Tukey’s test) (Fig. 5b). Additionally, differences between following species were not significant (*P* > 0.05, Tukey’s test).

### Multitrophic Effects

**Figure 6:**
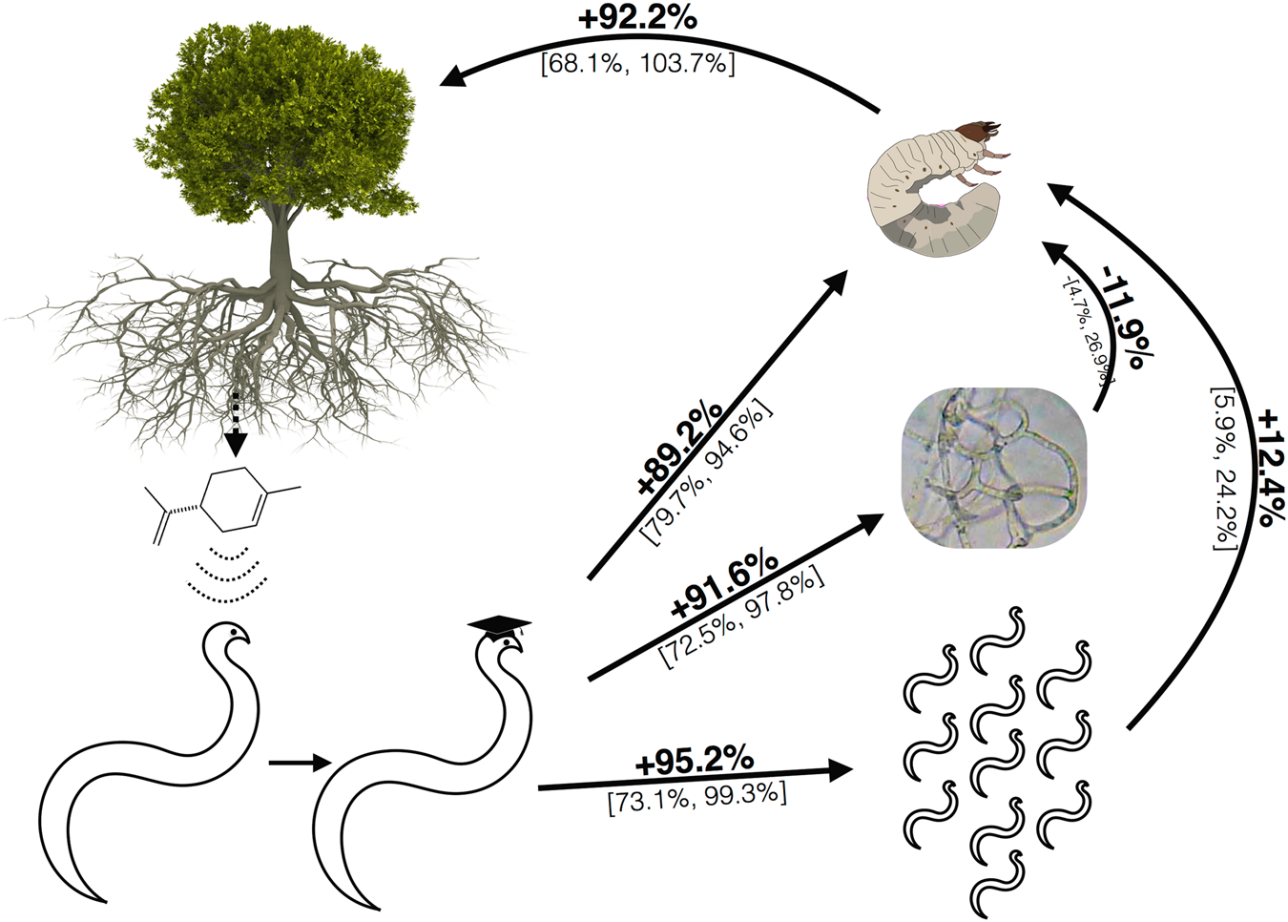
Multitrophic Effects of Belowground Parasitoid Learning. Plants release volatiles in response to induction of plant defense pathways. Exposure to these volatiles, in addition to recruiting entomopathogenic nematodes, can alter nematode infection behavior. Behavioral plasticity in response to plant volatiles can influence entomopathogenic nematode hosts, nematophagous fungal predators, and other entomopathogenic nematodes not exposed to the volatiles. These multitrophic effects of belowground entomopathogenic behavioral plasticity culminate in reducing root herbivory. Percent values indicate percent change in infection probability of *D*. *abbreviatus* in the presence of the plant volatile d-limonene due to behavioral plasticity of entomopathogenic nematode infective juveniles resulting from d-limonene exposure. Ranges in brackets indicate 95% confidence intervals. Arrows indicate direction of effect, not flow of energy as in traditional food web diagrams. Citrus tree ©Can Stock Photo Inc. Insect larva courtesy BugBoy 52.40. Fungal hyphae courtesy Bob Blaylock.

In examining cascading multitrophic effects, entomopathogenic nematode behavioral plasticity in response to d-limonene exposure directly increases host infection probability by 89.2% (Fig. 6, Table 1). Similarly, such behavioral plasticity results in an 91.6% increase in host infection probability in the presence of nematophagous fungi. The presence of nematophagous fungi, however, reduces probability of host infection by 11.9%. Increasing the pool of available responding infective juveniles (the social effect) increases probability of host infection by 95.2%. If leaders of responding infective juveniles have been exposed to d-limonene, probability of host infection increases by 12.4%. Averaging these effects across all three scenarios, exposure to the plant volatile d-limonene increases host infection probability by 92.2% (Fig. 6, Table 1).

**Table 1:** Multitrophic effects of behavioral plasticity in entomopathogenic nematodes.

Trial	Effect	Contrast	Change	95% CI	*P*
Host Effects	Learning	Exposure to d-limonene vs. Exposure to blank control	+89.2%	[79.7%, 94.6%]	<0.0001
Predator Effects	Learning	In the presence of *A*. *dactyloides*: Exposure to d-limonene vs. Exposure to blank control	+91.6%	[72.5%, 97.8%]	0.001
	Fungal Presence	Exposure to d-limonene: *A*. *dactyloides* presence vs. *A*. *dactyloides* absence	−11.9%	−[4.7%, 26.9%]	<0.0001
Social Effects: No 2	Following Behavior	50 + 2450 infective juveniles vs 50 infective juveniles	+95.2%	[73.1%, 99.3%]	0.003
	Learning	Exposure to d-limonene vs exposure to blank control	+12.4%	[5.9%, 24.2%]	<0.0001
Overall	Combined	Overall effect of exposure to the plant volatile d-limonene	+92.2%	[68.1%, 103.7%]	

Change reflects percent change in host infection probability. Overall effects are estimated by averaging effects from Host, Predator, and Social Effects trials.

## Discussion

Increases in host infection probability following entomopathogenic nematode exposure to plant volatiles suggests that behavioral plasticity may hold adaptive significance in organisms far removed from primates and New Caledonian crows. While entomopathogenic nematodes have limited neuronal capacity (*C*. *elegans*, for example, has a grand total of 302 neurons^28^) relative to the complex cerebral circuitry of such higher organisms, their demonstrated capacity for learning holds cascading implications for the rest of their belowground community.

First and foremost, behavioral plasticity in response to plant volatile exposure holds adaptive significance for those nematodes exposed to the volatiles themselves. The belowground environment is dynamic and punctuated by ephemeral signals released by plants, nematodes, and other organisms. Plant volatile responses to herbivory, for example, can peak at nine hours and disappear completely after twenty-four^29^. By taking advantage of brief exposures to plant volatiles, entomopathogenic nematodes may be able to avail themselves of transient resources (host larvae), which can become rapidly unsuitable for colonization due to competition from other nematodes^30–32^. While this form of behavioral plasticity is not associative in the classical sense, the first contact entomopathogenic nematodes have after leaving the cadaver may be a sufficient signal to alter future recruitment behavior. Additionally, changes in host infection probability after exposure to pregeijerene suggest that such behavioral plasticity is not limited to mere recruitment behavior. Indeed, changes in numbers of nematodes recruiting after exposure to pregeijerene are minimal (1.3% to 3.2%, *P* > 0.05)^26^. The large increases in host infection probability after exposure to pregeijerene suggest that infection behavior, such as willingness to enter the host larva, may be plastic as well.

Second, behavioral plasticity of entomopathogenic nematode parasitoids in response to plant volatile exposure negatively impacts host species. Natural hosts show increases in infection probability which would likely translate to increased mortality in the field. Additionally, other hosts were also negatively affected by entomopathogenic nematode behavioral plasticity in our investigation. Increases in mortality of other hosts demonstrate the potential for appropriating parasitoid learning capabilities for broadly enhancing biological control of multiple insect pests. While using exposed – ‘trained’ - parasitoids for control of traditional hosts will likely increase efficacy of biological control, the same strategies could be employed to enhance control of and target non-traditional hosts.

Third, in addition to affecting host infection probability, behavioral plasticity in entomopathogenic nematodes affects a higher trophic level: nematophagous fungal predators. Nematophagous fungi are thought to play a critical role in regulating entomopathogenic nematode populations and affect biological control using entomopathogenic nematodes in the field^32–34^. Behavioral plasticity of entomopathogenic nematodes allows them to overcome the nematophagous fungal gauntlet and infect their host insect larvae. Regulation of entomopathogenic nematode populations by nematophagous fungi benefits the insect host population while the ability of entomopathogenic nematodes to overcome such regulation through behavioral plasticity has negative consequences for both the host insect larvae and the nematophagous fungi which lose a food resource. The ability of entomopathogenic nematodes to overcome predation by nematophagous fungi after exposure to a plant volatile bears further investigation but could potentially be attributable to two mechanisms: (1) Learned responses to plant volatiles may motivate entomopathogenic nematodes to move faster, potentially making them harder to catch in fungal snares. (2) Conditioning of entomopathogenic nematodes to plant volatiles may alter the chemical ecology of nematode-fungal interactions. Increased response by entompathogenic nematodes to a given volatile after preexposure can be accompanied by a reduced response to other volatiles^26^. Training entomopathogenic nematodes to respond to a specific plant volatile may make them less likely to respond to other environmental stimuli, such as attractants released by nematophagous fungi^33, 35, 36^.

Fourth, behavioral plasticity in entomopathogenic nematodes affects other nematodes. The ability of nematodes to follow each other, even members of different genera, suggests a remarkable level of conserved communication across diverse species and genera that holds broad implications for the belowground community. Such following behavior might augment the evolutionary utility of ‘sprinter’ individuals^37^ as well as provide an explanation for observed aggregative movement in entomopathogenic^33, 38^ and other parasitic nematodes^39^. While this aggregative movement holds benefits for nematodes overcoming host immune systems, the implications for inter-specific competition within hosts remains to be explored. Given the persistence of conserved communication in entomopathogenic nematodes, we may find that social behavioral plasticity persists due to other benefits for multiple species within a given host. Additionally, social behavioral plasticity in entomopathogenic nematodes resulting in group movement based on a leader-follower dynamic may provide a means of signal amplification in belowground ecosystems.

From a plant perspective, this signal amplification is advantageous. A signal that reaches large numbers of parasitoids is costly and difficult in a patchy belowground environment. Instead, plants can benefit from releasing a brief ephemeral signal that reaches a few parasitoids (potentially pitifully few, not even enough to infect a single herbivore) which is then amplified by the social behavioral plasticity of belowground entomopathogenic nematode parasitoids. Ultimately, social behavioral plasticity of belowground parasitoids in response to exposure to plant volatiles results in a net 93.2% increase in probability of host infection (Fig. 6). By exposing entomopathogenic nematodes to specific volatiles, plants can almost double the likelihood of infecting and eventually killing insect larval herbivores.

The adaptive significance of signal amplification by belowground behavioral plasticity highlights the importance of plants and their defense pathways (which produce the signals) as key regulators of community structure^40^. Additionally, the cascade of multitrophic effects from social behavioral plasticity suggests an equally important role for behavioral plasticity in shaping community dynamics. Further understanding these effects, and their interactions with plant signaling, will enhance our ability to efficiently and efficaciously manage above and belowground natural systems.

## Material and Methods

### Host Effects

To determine the effects of learning in entomopathogenic nematode parasitoids on their hosts, infection of *Diaprepes abbreviatus* weevil larvae was evaluated with cohorts of exposed and non-exposed entomopathogenic nematodes. Cohorts of 2500 *Steinernema diaprepesi*, *Steinernema riobrave*, *Steinernema carpocapsae*, or *Heterorhabditis indica* infective juveniles were placed into 5 *ml* water solutions. Nematode cohort solutions then received 5 *μl* of pentane (blank control) or 5 *μl* of 1 *μg*/*μl* of either pregeijerene, d-limonene, or (*E*)-*β* caryophyllene in pentane. Nematodes remained in respective exposure solutions for 72 hours. After 72 hours, nematode cohorts were washed with DI water and released into the central chamber of a four-arm, sand-filled glass olfactometer.

Four-arm glass olfactometers were constructed from traditional six-arm glass olfactometers used in previous trials^14, 15^ by closing off two of the six arms. Glass olfactometers were further modified by removing all mesh screens to allow entomopathogenic nematodes access to all parts of the olfactometer. Swingle citrumelo (*Citrus paradisi* Macf. x *Poncirus trifoliata* L. Raf.) rootstocks were placed in the distal portion of all four arms of the olfactometer along with a single fifth instar *D*. *abbreviatus* larva encaged in a stainless steel 1 *cm* ^2^, 225 mesh screen cube. This mesh size allowed entomopathogenic nematodes to enter and infect larvae, but prevented larvae from feeding on citrus roots. Aliquots consisting of either 30 *μl* pentane (blank control) or 30 *μl* of 10 *ng*/*μl* solution of pregeijerene, d-limonene, or (*E*)-*β* caryophyllene in pentane were placed on filter paper and added to the root zone in each of the four arms. The olfactometer was then filled with washed autoclaved sand adjusted to 10% moisture by volume. Twenty-four hours after introduction of the entomopathogenic nematode infective juveniles, *D*. *abbreviatus* larvae were collected from each arm and placed on White traps^41^ to monitor for infection. Twenty replications of each exposure cohort-nematode species combination were conducted.

### Additional Host Effects

To determine the effects of learning in entomopathogenic nematodes on hosts other than *D*. *abbreviatus*, cohorts of exposed and non-exposed *H*. *indica* infective juveniles were presented to larvae of the weevil *D*. *abbreviatus* (host), the waxworm *Galleria mellonella* (used for rearing entomopathogenic nematodes), the beetle *Tenebrio molitor*, and the Caribbean fruit fly *Anastrepha suspensa*. As in the host effect trials, cohorts of 2500 *Heterorhabditis indica* infective juveniles were placed into 5 *ml* water solutions. Nematode cohort solutions then received 5 *μl* of pentane (blank control) or 5 *μl* of 1 *μg*/*μl* of d-limonene in pentane. Nematodes remained in respective exposure solutions for 72 hours. After 72 hours, nematode cohorts were washed with DI water and released into the central chamber of a four-arm, sand-filled glass olfactometer. Monitoring infection of larvae in olfactometers was conducted as described in Host Effects, with substitutions of alternative hosts for *D*. *abbreviatus* larvae. Twenty replications of each exposure cohort-nematode species combination were conducted.

### Predator Effects

To explore effects of nematophagous fungi on host infection by entomopathogenic nematodes, cohorts of *H*. *indica* infective juveniles were exposed to either a blank control or d-limonene for 72 hours as in Host Effects trials. After exposure, the cohorts were introduced into glass four-choice, sand-filled olfactometers whose central chambers were either inoculated 24 hours prior with the nematophagous fungus *Arthrobotrys dactyloides* or a blank control. Arrayed around the central chamber were insect (*G*. *mellonella*) larvae paired with either a blank control, d-limonene, (*E*)-*β* caryophyllene, or pregeijerene as described above. After allowing 24 hours for infection, insect larvae (*G*. *mellonella* in this case) were removed and monitored for infection. Twenty replications of each exposure cohort-fungal presence combination were conducted.

### Host Effects of Social Parasitoid Learning

To explore effects of social behavioral plasticity in entomopathogenic nematodes on host infection, cohorts of 50 *H*. *indica* infective juveniles were exposed to either a blank control or d-limonene for 72 hours as described in Host Effects above. After exposure, the cohorts of 50 exposed *H*. *indica* were combined with cohorts of 2450 nonexposed infective juveniles of either *H*. *indica*, *S*. *riobrave*, or *S*. *diaprepesi* and introduced to glass four-choice, sand-filled olfactometers as described above. The infective juveniles were given twenty-four hours in which to infect *D*. *abbreviatus* larvae paired with either a pentane blank control, d-limonene, (*E*)-*β* caryophyllene, or pregeijerene as described above. Host infection from these mixed cohorts was then contrasted with infection resulting from cohorts consisting of 2500 infective juveniles of either *H*. *indica*, *S*. *riobrave*, *S*. *diaprepesi* not exposed to any plant volatiles.

To further explore abilities of small numbers of infective juveniles to affect larger populations, host infection by cohorts consisting of either 2500 infective juveniles exposed to d-limonene, mixed cohorts consisting of 50 *H*. *indica* infective juveniles exposed to d-limonene and 2450 infective juveniles not exposed to plant volatiles, or 2500 infective juveniles not exposed to d-limonene were compared using bioassay procedures described above for both *H*. *indica* and *S*. *diaprepesi*.

### Organisms

All four species of entomopathogenic nematodes (*Steinernema diaprepesi*, *Steinernema riobrave*, *Steinernema carpocapsae*, or *Heterorhabditis indica*) were reared in late instar *Galleria mellonella* waxworm larvae^42^. After emergence from infected host carcasses, entomopathogenic nematode infective juveniles were collected on White traps, transferred to tissue culture flasks, and stored at 10 °C until use^41^. All infective juveniles used in experimental trials were less than two weeks old.

*D*. *abbreviatus* larvae were reared on artificial diet from eggs laid by adults collected from Citrus groves in Polk County, FL, USA following procedures outlined in previous work^43, 44^. Waxworm *G*. *mellonella* larvae were obtained from Speedy Worm and Minnesota Muskie Farms Inc. *T*. *molitor* larvae were obtained from Fluker Farms, Port Allen, LA. *A*. *suspensa* larvae were obtained from a long-established culture maintained by N. Epsky (Subtropical Horticulture Research, United States Department of Agriculture-Agricultural Research Service, Miami, FL).

*A*. *dactyloides* nematophagous fungi were originally isolated from citrus groves in Polk County, FL, USA then cultured at the Citrus Research and Education Center (CREC) in Lake Alfred, FL on quarter strength corn meal agar^17^. All fungi were sub-cultured monthly and maintained on agar within 9-cm diameter Petri dishes at 27.5 °C. Fungi were exposed to living *S*. *diaprepesi* 21 d prior to initiation of assays by spreading approximately 3,000 sterilized (0.2% streptomycin sulphate exposure for 24 h according to methods of ref. 33) IJs over fungal cultures on plates. Sand was inoculated with fungi 14 d prior to assays with ten 1-cm diameter agar disks collected from IJ-exposed fungal cultures growing on Petri dishes. Discs were gently placed onto sand and buried in sequential layers throughout the glass pots comprising the distal ends of the radiating arms of the olfactometer. The 10 discs were inserted into the sand equidistantly from the bottom up with approximately 0.5 cm of sand in between individual discs throughout the sand column within each pot.

Swingle citrumelo (*Citrus paradisi* Macf. × *Poncirus trifoliata* L. Raf.) rootstocks were grown and maintained at the CREC in a greenhouse at 26 °C and 60–80% RH^15^.

### Statistical Analysis

Host infection (binary: either infected or not infected) by entomopathogenic nematodes was modeled using logistic regression (generalized linear models based on a binomial distribution with a logit link function). Model selection was accomplished through examination of analysis of deviance, psuedo *R* ^2^ values^45^, *χ* ^2^ tests of log likelihood, and Bayesian information criteria. Host infection probabilities for given treatment combinations, along with estimates of uncertainty, were then reported from model predictions. Post-hoc contrasts adjusting for *α*-inflation were accomplished using Tukey’s and Dunnett’s tests.

Overall net effects of behavioral plasticity on host infection probability were conservatively estimated by averaging means, lower 95% confidence levels, and upper 95% confidence intervals across trophic levels.

### Computing Environment

Data were collected and collated in Microsoft Excel then analyzed using R version 3.3.1 in the RStudio development environment version 0.99.902. Additional packages were used to facilitate analysis. *readxl* ^46^ was used for Excel-R communication. *dplyr* ^47^ and *tidyr* ^48^ were used for data formatting, *car* ^49^ for analysis of deviance, *lsmeans* ^50^ for post-hoc comparisons and *ggplot*2^51^ with *scales* ^52^ for elegant graphics.
